# Abnormal uterine bleeding during menopausal hormone therapy: Number 8 – 2026

**DOI:** 10.61622/rbgo/2026FPS8

**Published:** 2026-06-18

**Authors:** Ana Lúcia Ribeiro Valadares, Luiz Francisco Cintra Baccaro, Lúcia Helena Simões da Costa-Paiva, Elizabeth Jeha Nasser, Elvira Maria Mafaldo Soares, Marco Aurélio Albernaz, Mario Vicente Giordano, Eliana Aguiar Petri Nahas, Jaime Kulak, Luciano de Melo Pompei, Márcio Alexandre Hipólito Rodrigues, Maria Celeste Osório Wender, Maria Célia Mendes, Mona Lucia Dall’Agno, Rita de Cassia Dardes, Rodolfo Strufaldi

**Affiliations:** Universidade Estadual de Campinas Campinas SP Brazil Universidade Estadual de Campinas, Campinas, SP, Brazil.; Universidade Estadual de Campinas Campinas SP Brazil Universidade Estadual de Campinas, Campinas, SP, Brazil.; Universidade Estadual de Campinas Campinas SP Brazil Universidade Estadual de Campinas, Campinas, SP, Brazil.; Faculdade de Medicina do ABC Departamento de Ginecologia e Obstetrícia Santo André SP Brazil Departamento de Ginecologia e Obstetrícia, Faculdade de Medicina do ABC, Santo André, SP, Brazil.; Universidade Federal do Rio Grande do Norte Brazil Universidade Federal do Rio Grande do Norte, Brazil.; Universidade Federal de Goiás Goiânia Brazil Universidade Federal de Goiás, Goiânia, Brazil.; Universidade Federal do Estado do Rio de Janeiro Rio de Janeiro RJ Brazil Universidade Federal do Estado do Rio de Janeiro, RJ, Rio de Janeiro, Brazil.; Universidade Estadual Paulista "Júlio de Mesquita Filho" Botucatu SP Brazil Universidade Estadual Paulista "Júlio de Mesquita Filho", Botucatu, SP, Brazil.; Universidade Federal do Paraná Curitiba PR Brazil Universidade Federal do Paraná, Curitiba, PR, Brazil.; Faculdade de Medicina do ABC Santo André SP Brazil Faculdade de Medicina do ABC, Santo André, SP, Brazil.; Universidade Federal de Minas Gerais Belo Horizonte MG Brazil Universidade Federal de Minas Gerais, Belo Horizonte, MG, Brazil, GO, Brazil.; Universidade Federal do Rio Grande do Sul Porto Alegre RS Brazil Universidade Federal do Rio Grande do Sul, Porto Alegre, RS, Brazil.; Universidade de São Paulo Faculdade de Medicina de Ribeirão Preto São Paulo Ribeirão Preto Brazil Faculdade de Medicina de Ribeirão Preto, Universidade de São Paulo, Ribeirão Preto, São Paulo, Brazil.; Universidade de Caxias do Sul Caxias do Sul RS Brazil Universidade de Caxias do Sul, Caxias do Sul, RS, Brazil.; Universidade Federal de São Paulo Escola Paulista de Medicina São Paulo SP Brazil Escola Paulista de Medicina, Universidade Federal de São Paulo, São Paulo, SP, Brazil.; Faculdade de Medicina do ABC Departamento de Ginecologia e Obstetrícia Santo André SP Brazil Departamento de Ginecologia e Obstetrícia, Faculdade de Medicina do ABC, Santo André, SP, Brazil.

## Key points

- Unscheduled bleeding is considered an undesirable side effect and one of the main reasons for discontinuation of menopausal hormone therapy (MHT).
- Bleeding in the first few months of MHT is expected, especially in continuous regimens, but persistence after six months or bleeding after a period of amenorrhea requires further investigation.
- The pathophysiology of abnormal uterine bleeding (AUB) is multifactorial; underlying factors include hormonal, vascular and functional changes in the endometrium.
- The prevalence of endometrial atrophy as a cause of AUB during MHT is significant, especially in postmenopausal women using continuous combined hormone therapy.
- Risk stratification for endometrial cancer should consider major factors (body mass index [BMI] ≥ 40 kg/m^2^, genetic syndromes, prolonged use of estrogen alone, and inadequate progestogen regimens) and minor factors (BMI between 30 and 39 kg/m^2^, diabetes, and polycystic ovary syndrome). Investigation is indicated in the presence of one major factor or two minor factors.

## Recommendations

- Transvaginal ultrasound is frequently used to rule out structural or malignant causes of bleeding.
- When choosing a hormone therapy regimen, it is important to consider long-term endometrial safety.
- The co-administration of a sequential progestogen is recommended to ensure safety of non-hysterectomized women initiating estrogen replacement therapy.
- The regimen (sequential or continuous), duration of use, route of administration, and patient risk profile should be considered when individualizing MHT.
- Management of AUB should be individualized and phased, starting with optimization of hormone therapy (dose adjustment, route change, alteration of the type or duration of progestogen) and progressing to regimen change (sequential to continuous or vice versa) or to the use of a levonorgestrel-releasing intrauterine device (LNG-IUD), when necessary.

## Background

Hormone therapy is considered the most effective treatment for peri- and postmenopausal symptoms. Despite the various benefits associated with menopausal hormone therapy (MHT), unscheduled uterine bleeding is frequent among women using combined estrogen-progestogen therapy. Both sequential and continuous combined MHT regimens were developed to ensure predictable bleeding or the total absence of bleeding. Thus, unscheduled bleeding is considered an undesirable side effect and constitutes one of the main reasons for abandoning therapy, affecting up to one-third of users. Its frequency and intensity depend on the regimen, formulation, dose, route of administration and duration of hormone use.^(1,2)^

## What are the pathophysiological mechanisms related to abnormal uterine bleeding (AUB) in MHT users?

The pathophysiology of AUB is multifactorial. Hormonal, vascular and functional alterations of the endometrium are the underlying factors and depend on the regimen, formulation, and route of administration of MHT.

### Endometrial vascularization

The use of estrogens and progestogens changes the density, distribution, and structure of endometrial blood vessels, increasing vascular fragility and susceptibility to bleeding. Combined MHT can lead to disordered angiogenesis, resulting in thin vascular walls and increased permeability, which favors blood extravasation. Furthermore, MHT increases the production of prostaglandins, which can worsen vascular instability.^(3,4)^

### Stroma and extracellular matrix

Menopausal hormone therapy can cause changes in the endometrial stroma, such as remodeling of the extracellular matrix and increased activity of metalloproteinases, enzymes responsible for tissue degradation. This can lead to fragile areas and increase the risk of bleeding, especially in MHT regimens that do not favor decidualization or endometrial atrophy.^(5,6)^

In women with AUB during MHT, there is an increase in the expression of metalloproteinases, such as MMP-3, MMP-9 and their inhibitors, in addition to a greater presence of uterine natural killer (NK) cells, indicating that matrix degradation and immune activation may contribute to vascular rupture and bleeding.^(7)^

### Imbalance between cell proliferation and apoptosis

The balance between proliferation, differentiation, and apoptosis of endometrial cells is crucial for tissue integrity. Estrogen stimulates proliferation, while progestogen favors differentiation and decidualization. In MHT, especially in continuous combinations, imbalances between these processes may occur due to inadequate doses of estrogen and progestogen, resulting in persistent proliferative areas or incomplete decidualization, which may contribute to AUB.^(5,8)^

### Hormonal deprivation

A precipitous decline in serum levels of estrogen and progestogen (in continuous combined regimen) or progestogen alone (in sequential regimen) triggers endometrial shedding, mirroring the physiological process of the natural menstrual cycle.^(6,9)^

## What is the diagnostic approach for AUB during MHT?

Structural or malignant causes of bleeding, such as polyps, adenomyomas, leiomyomas, malignancy, and hyperplasia should be ruled out. Transvaginal ultrasound is frequently used to assess the presence of structural etiologies.^(10)^ Risk factors for endometrial cancer should be considered and classified as major and minors (Chart 1).^(11–13)^

**Chart 1:** Risk factors for endometrial cancer

Major Risk Factors	Minor risk factors
BMI ≥ 40 kg/m^2^	BMI between 30 and 39 kg/m^2^
Associated genetic syndromes (Lynch or Cowden)	Use of estrogen alone for more than 3 months and less than 6 months
Use of estrogen alone for more than 6 months in women with a uterus	MHT with progesterone every 3 months for more than 6 months and less than 1 year
MHT with progesterone administered every 3 months for more than 1 year	During 6 to 12 months, use of norethisterone or MPA for less than 10 days/month or micronized progesterone for less than 12 days/month in a sequential regimen
Sequential MHT for more than 5 years in women who started treatment at ≥ 45 years of age	Inadequate progestogen dose relative to estrogen dose for more than 1 year
Use of norethisterone or MPA for less than 10 days/month or micronized progesterone for less than 12 days/month in a sequential regimen for ≥ 12 months	Anovulatory cycles, such as in polycystic ovary syndrome
	Diabetes mellitus

BMI: body mass index; MHT: menopausal hormone therapy; MPA: medroxyprogesterone acetate

The presence of one major risk factor or two minor risk factors requires investigation, especially in cases of heavy or persistent bleeding. In women with endometrial thickness (ET) on transvaginal ultrasound using continuous combined regimens with ET > 4 mm and sequential combined regimens with ET > 7 mm, endometrial biopsy should be performed, preferably guided by hysteroscopy.^(13)^ Incomplete visualization of the endometrial line is also a criterion for further investigation with histology.^(13,14)^ If the initial assessment is normal and there is no high risk, it is recommended to optimize hormone therapy and observe the patient. If bleeding persists, endometrial biopsy is indicated for diagnosis. This approach seeks to balance the prevention of unnecessary procedures with the early diagnosis of malignant conditions.^(13)^

## How to treat AUB during MHT?

It is important to consider long-term endometrial safety when choosing a hormone therapy regimen. Sequential co-administration of a progestogen is recommended for non-hysterectomized women initiating estrogen replacement therapy to ensure safety.^(15)^ In cases of unscheduled bleeding, a rigorous clinical assessment is essential to exclude endometrial malignancy, particularly given the elevated risk of endometrial adenocarcinoma in postmenopausal women. However, evidence suggests that after investigating AUB in patients on hormone therapy, endometrial atrophy (ET < 4 mm) is identified in up to 60% of cases where structural pathology or hyperplasia is absent; this remains the primary etiology of benign bleeding in this population. Furthermore, factors such as medication non-adherence, inconsistent dosing schedules, and potential drug interactions are significant contributors to bleeding during MHT.^(3,5)^ A systematic review suggests that oral hormone therapy formulations have a higher bleeding profile than transdermal formulations.^(4)^ Cumulative amenorrhea over one year ranged from 18% to 61% with oral hormone therapy and from 9% to 27% with transdermal therapy.^(4)^ For example, the oral combination of estradiol and progesterone is associated with lower bleeding rates and may be an appropriate alternative for women seeking bioidentical hormone therapy or who have concerns about bleeding with other hormone therapies.^(4)^

The management of AUB during sequential estrogen-progestogen therapy should follow a systematic approach, considering both structural and functional causes. Initially, it is essential to exclude structural causes (such as polyps, fibroids, endometrial hyperplasia or cancer) and other non-hormonal etiologies, especially in women with risk factors or age ≥ 45 years through detailed medical history, physical examination, transvaginal ultrasound and, if indicated, endometrial sampling.^(10)^

If structural causes and malignancy are excluded, bleeding can be considered a side effect of the sequential therapy itself. Intermenstrual or irregular bleeding is relatively common, especially in the first few months of use, and may be related to the type, dose, or regimen of progestogen and estrogen used.^(15,16)^

Since there is no absolute correlation between the bleeding pattern and endometrial histology, persistent bleeding requires further investigation.^(15)^

Adjustments to therapy may be considered, such as modifying the progestogen dose, increasing the duration of progestogen use in the sequential phase (e.g., from 10 to 12-14 days per cycle), or changing the type of progestogen or estrogen, always monitoring the clinical response.^(17)^

In cases of persistent bleeding, switching to a continuous combined regimen may be considered, as it tends to reduce bleeding episodes after the initial adaptation period.^(18)^

Abnormal uterine bleeding during continuous estrogen-progestogen therapy is more common in the first few months of use, with a tendency towards amenorrhea over time, but persistent episodes or late bleeding requires further investigation to exclude organic causes, especially endometrial malignancy.^(18,19)^

Initially, it is essential to assess the time of onset of bleeding in relation to the start of therapy. Bleeding in the first 3-6 months is frequent and usually self-limiting, especially with combined formulations of estradiol and norethisterone acetate or medroxyprogesterone, being more common at lower doses of progestogen and in transdermal formulations and rarer at higher doses of progestogen.^(18,19)^

Patients should be advised that the incidence of bleeding decreases over time.^(18)^ If bleeding persists after the first six months of use or occurs after a period of amenorrhea, diagnostic investigation is recommended to exclude endometrial hyperplasia or cancer. This includes gynecological examination, transvaginal ultrasound for evaluation of ET and, if indicated, endometrial biopsy.^(4,19)^

In cases of persistent bleeding in patients without abnormalities, adjustments to hormone therapy may be considered, such as increasing the dose of progestogen, changing the formulation (e.g., from transdermal to oral) or switching to a sequential regimen, especially if the bleeding pattern is intolerable to the patient.^(4,19)^

In situations of chronic irregular bleeding, the literature suggests that reversion to a sequential regimen may be appropriate.^(19)^

In sequential MHT:

- If the patient is perimenopausal, < 50 years old, requires contraception, and has a low thrombotic risk, consider switching from MHT to a combined oral contraceptive (COC) — COC with 1.5 mg estradiol and a progestogen.
- Switch to oral MHT.
- Increase the dose of micronized progesterone (300 mg for 14 days) or switch to 10 mg norethisterone or dydrogesterone.
- Offer a 52 mg levonorgestrel-releasing intrauterine device (LNG-IUD).
- Three-month trial with additional progestogen (100 mg micronized progesterone [MP], 10 mg medroxyprogesterone acetate, or 5 mg norethisterone [NET] for three months).^(13)^

In continuous MHT:

- Consider switching to the oral route, if possible.
- Reduce the estrogen dose, as lower doses are associated with a better bleeding pattern.
- Increase the dose or change the type of progestogen, or switch to a 52 mg LNG-IUD (or if the user switches after four years) — (MP to 200 mg per day or 1.0 mg NET or 10 mg dydrogesterone).
- Recommend estrogen alone for three months, followed by progestogen for 14 days, and return to the continuous estradiol + progesterone regimen (risk of endometrial cancer).
- Finally, switch to sequential MHT.^(13)^

## Discussion

The document emphasizes that AUB during hormone therapy represents a significant clinical challenge, substantially affecting treatment adherence.^(1,2)^ Understanding the underlying pathophysiological mechanisms is fundamental for appropriate and individualized management. The multifactorial nature of AUB in MHT users is highlighted, involving complex interactions between vascular changes, tissue remodeling, hormonal imbalance, and immune response. The increased expression of metalloproteinases and the presence of uterine NK cells in women with AUB suggest that inflammatory processes and extracellular matrix degradation play an important role.^(3–9)^

Risk stratification based on major and minor factors allows for a more rational and cost-effective approach.^(10–12)^

**Figure 1:**
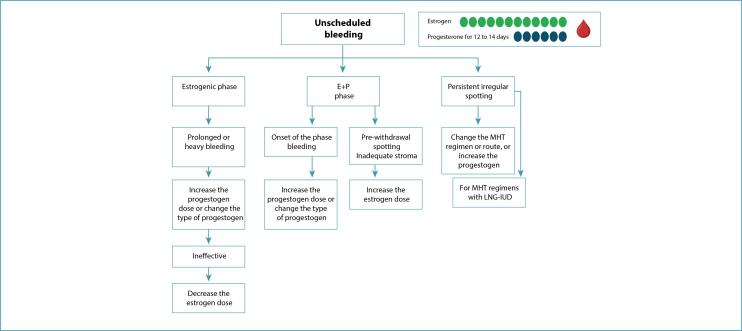
Algorithm for managing bleeding in sequential MHT

MHT: Menopausal hormone therapy; HT: Hormone therapy; LNG-IUD: Levonorgestrel-releasing intrauterine device

Source: Adapted from Manley et al. (2024).^(13)^

The individualized management of AUB is key, considering the duration of MHT use (bleeding in the first few months is expected), the type of regimen (sequential vs. continuous), the route of administration (oral vs. transdermal), the patient's risk profile, and individual tolerance and preferences (Figure 1).^(13)^

The evidence presented demonstrates the superiority of oral formulations over transdermal ones in controlling bleeding, highlighting the combination of estradiol and oral progesterone as an option with lower bleeding rates.^(13)^

The document proposes a phased approach to management, starting with simpler adjustments (route change, dose adjustment) and progressing to more complex interventions (regimen change, LNG-IUD) as needed, always after excluding structural and malignant causes.^(13)^

## Final considerations

The approach to MHT should balance effectiveness in controlling menopausal symptoms, minimizing bleeding, and long-term endometrial safety, with regular clinical monitoring and adjustments as needed.

## Data Availability

the research data are available in the article.

## National Specialized Commission on Menopause of the Brazilian Federation of Gynecology and Obstetrics Associations (FEBRASGO)

President:

Lucia Helena Simoes da Costa Paiva

Vice-president:

Luciano de Melo Pompei

Secretary:

Eliana Aguiar Petri Nahas

Members:

Mona Lúcia Dall’Agno
Elvira Maria Mafaldo Soares
Elizabeth Jeha Nasser
Ana Lucia Ribeiro Valadares
Mario Vicente Giordano
Jaime Kulak Junior
Luiz Francisco Cintra Baccaro
Márcio Alexandre Hipólito Rodrigues
Marco Aurélio Albernaz
Maria Celia Mendes
Rita de Cassia de Maio Dardes
Rodolfo Strufaldi
